# The Effects of Diet and Dietary Interventions on the Quality of Life among Breast Cancer Survivors: A Cross-Sectional Analysis and a Systematic Review of Experimental Studies

**DOI:** 10.3390/cancers12020322

**Published:** 2020-01-30

**Authors:** Martina Barchitta, Andrea Maugeri, Roberta Magnano San Lio, Annalisa Quattrocchi, Flori Degrassi, Francesca Catalano, Guido Basile, Antonella Agodi

**Affiliations:** 1 Department of Medical and Surgical Sciences and Advanced Technologies “GF Ingrassia”, University of Catania, Via S. Sofia 87, 95123 Catania, Italy; martina.barchitta@unict.it (M.B.); andrea.maugeri@unict.it (A.M.); robimagnano@gmail.com (R.M.S.L.); 2 Department of Primary Care and Population Health, University of Nicosia Medical School, 21 Ilia Papakyriakou, 2414 Engomi, Nicosia, Cyprus; quattrocchi.a@unic.ac.cy; 3 Associazione Nazionale Donne Operate al Seno A.N.D.O.S. onlus nazionale, 20154 Milan, Italy; flori.degrassi@libero.it; 4 Multidisciplinary Breast Unit, Azienda Ospedaliera Cannizzaro, 95126 Catania, Italy; dr.francescacatalano@virgilio.it; 5 Department of General Surgery and Medical-Surgical Specialties, University of Catania, Via Plebiscito 628, 95124 Catania, Italy; gbasile@unict.it

**Keywords:** breast cancer, quality of life, public health, diet, nutrition, dietary intervention, physical activity, exercise

## Abstract

There is an ongoing need for solid evidence about the effects of healthy behaviors, and particularly diet, on the quality of life (QoL) among breast cancer survivors. We first conducted a cross-sectional study on 68 Italian stage I-III breast cancer survivors, to investigate the association of adherence to the Mediterranean diet (MD), physical activity and weight status with QoL. Adherence to MD and physical activity was assessed using structured questionnaires. QoL was assessed using the European Organization for the Research and Treatment of Cancer Quality-of-Life tools. We showed that low consumption of red meat and carbonated beverages, daily consumption of wine and high consumption of dishes seasoned with sofrito had beneficial effects on several QoL subscales. By contrast, using olive oil as the main culinary fat, low consumption of commercial sweets and high consumption of nuts were associated with negative effects. Overall, these findings resulted in a null effect of adherence to MD on QoL. Furthermore, we observed better QoL sub-scores among women who performed moderate physical activity (i.e., diarrhea) and those who were underweight/normal weight (i.e., physical functioning and dyspnea) if compared with their counterparts (*p*-values ≤ 0.003 after correction for multiple comparison). Next, we performed a systematic review of nine experimental studies to summarize whether dietary interventions might improve QoL among breast cancer patients. All the studies demonstrated significant improvements in overall QoL and/or its subscales after the interventions. However, differences in study design, interventions and tools used for QoL assessment did not allow us to provide an overall estimate. Moreover, only a single-arm trial evaluated the effect of an exclusive dietary-based intervention, while others combined dietary recommendations with physical activity and weight loss programs. For these reasons, our study encourages more efforts to improve the robustness of current evidence, through more homogenous tools, larger population-based studies and further randomized controlled trials.

## 1. Introduction

Breast cancer is the most common cancer among women worldwide [1], with an incidence that increased over the past decades, accounting for 2.09 million cases and 627.000 deaths in 2018 [2]. Recent strides in breast cancer diagnosis and treatment ameliorated patients’ care [3], which resulted in a growing number of survivors in developed countries [4]. However, breast cancer survivors exhibit an increased risk for secondary tumors, cardiovascular disease, diabetes [5,6,7] and reduced quality of life (QoL) [8,9]. Considering that breast cancer and its treatments are also associated with adverse effects [10,11], the survivors represent an important target population for promoting prevention strategies [12].

In general, the American Cancer Society (ACS) guidelines for cancer survivors recommend to consume vegetables, fruits and whole grains regularly [13], and previous evidence showed that the promotion of a healthy diet might reduce the risk of recurrence [14], stress and breast/arm symptoms [15]. Specifically, breast cancer patients showed reduced QoL than their healthy counterpart, with several dietary problems including dyspepsia, nausea and anorexia [16]. Changes in health related factors were evident among breast cancer survivors [17], but controversy exists about the association between diet and QoL. For instance, Orchard and colleagues did not demonstrate correlations between the healthy eating index score and QoL [18]. By contrast, Tangney and colleagues reported a negative correlation with depression, assessed by the Center for Epidemiologic Studies Depression scale (CES-D) score [19]. Others reported better global health status [20,21] and social, emotional, cognitive and role functioning [21], lower risk of dyspnea [22], but higher risk of insomnia [22] among breast cancer survivors with healthy dietary habits. For these reasons too, a previous study reported that 9 out of 10 women asked for a personalized dietary counseling after a breast cancer diagnosis [16]. However, observational evidence showed that dietary interventions did not modify dietary habits, with no significant changes in fruit or vegetable servings per day or take-away and fast food frequency per week [23].

Beyond dietary habits, the ACS guidelines for cancer survivors also recommend to achieve and maintain a healthy weight and to engage in regular physical activity. Accordingly, several lines of evidence from observational studies underlined the role of weight management and exercise to improve health status and QoL of breast cancer survivors [24,25,26,27,28]. Moreover, it has been demonstrated that a multidisciplinary rehabilitation program, based on occupational and physical counseling, supported a return to work by reducing fatigue and increasing working ability and QoL [29]. A trend analysis by Blanchard and colleagues showed a positive association between the number of recommendations and QoL in breast cancer survivors [12], which, however, was limited to those with stage II or III cancer [24]. In line, Lee and colleagues stated that increasing adherence to the ACS guidelines was positively associated with several QoL subscales [16]. Yet, recent population-based studies [30,31,32] in the United States and Australia have reported that up to 70% of cancer survivors ignore these recommendations.

Given the above, there is still the need for solid evidence about the effects of healthy behaviors, and particularly diet, on QoL among breast cancer survivors. Here, we first conducted a cross-sectional study to examine the association of adherence to the Mediterranean diet (MD), physical activity and weight status with the QoL of Italian breast cancer survivors. Next, we performed a systematic review of experimental studies to summarize whether dietary interventions, alone or in combination with physical activity recommendations, might significantly improve QoL among women with a breast cancer diagnosis. 

## 2. Materials and Methods

### 2.1. Cross-Sectional Study

#### 2.1.1. Study Design

From 2013 to 2014, we conducted a cross-sectional study on Italian women who were diagnosed with stage I-III breast cancer and who completed radiotherapy or chemotherapy treatment at least 6 months prior to the recruitment. We excluded those who were diagnosed with stage 0 breast cancer or those who had breast cancer treatment within 6 months before recruitment. On a total of 162 invited women who met these criteria, 42% (*n* = 68) completed the assessment of behavioral and dietary data, anthropometric measures and QoL. The recruitment phase was supported by A.N.D.O.S. Onlus (Associazione Nazionale Donne Operate al Seno). The study protocol was approved by the ethical committees of the involved institutions (47/2014/CA), all women signed an informed consent, and the study was conducted according to the Declaration of Helsinki.

A structured questionnaire designed ad-hoc was administered to collect information on age, lifestyle characteristics (i.e., adherence to MD and physical activity) and self-reported anthropometric measures. Self-reported anthropometric measures were used to calculate and categorize body mass index (BMI) according to the World Health Organization (WHO) criteria [33]. Physical activity level was assessed using the long form of the International Physical Activity Questionnaire (IPAQ-L) [34], and categorized as low (no moderate and vigorous activities), moderate (1–149 min/week moderate or 1–74 min/week vigorous or 1–149 min/week moderate + vigorous), or high (≥150 min/week moderate or ≥75 min/ week vigorous or ≥150 min/ week moderate + vigorous) according to the American Heart Association recommendations [35]. 

#### 2.1.2. Dietary Assessment

The adherence to the MD was assessed using the Mediterranean Diet Assessment Tool proposed by Martinez-Gonzalez and colleagues [36,37,38,39]. This tool, developed in a Spanish case-control study of myocardial infarction [40], includes 14 items and criteria used for rating each component are reported in Tables S1–S3. Based on the overall score, the adherence to MD was categorized as low (≤5 positive items), medium (6–9 positive items) or high (≥10 positive items).

#### 2.1.3. Assessment of Quality of Life

The assessment of functional status and global QoL was performed using the European Organization for the Research and Treatment of Cancer Quality-of-Life (EORTC) Questionnaire–Core 30 (QLQ-C30) [41]. In brief, the QLQ-C30 includes the global health status/quality of life, the functional and the symptom scales [41]. Moreover, QoL assessment was accompanied by the administration of the Quality of Life Questionnaire Breast Cancer Module 23 (QLQ-BR23) [42,43]. The QLQ-BR23 is composed by 23 items organized in functional and symptom scales. The raw scores of the 4-point or 7-point scales were transformed to a 0–100 scale based on the EORTC scoring manual, with a higher score reflecting better QoL in functioning and global health status/quality of life and a worse QoL in symptoms [42,43].

#### 2.1.4. Statistical Analysis

The SPSS software (version 21.0, SPSS, Chicago, IL, USA) was used to perform all the statistical analyses. Continuous variables were tested for normality using the Kolmogorov–Smirnov test, reported as mean and standard deviation (SD), and compared using the Student’s *t*-test for comparison between two groups or the one-way ANOVA for comparison between more than two groups. All statistical tests were two-sided, and *p*-values < 0.05 were considered statistically significant. We also reported statistically significant results after Bonferroni correction (*p*-values ≤ 0.003 for QLQ-C30 and ≤ 0.006 for QLQ-BR23).

### 2.2. Systematic Review

#### 2.2.1. Literature Search and Study Selection

Two of the authors (A.M. and R.M.S.L.) independently conducted a literature search in the PubMed-Medline and Web of Science databases from the inception to May 2019, using the following MeSH terms: “Breast Cancer” and “Quality of Life” and (“Diet” or “Exercise”). Studies were included if they were consistent with the following criteria: (1) experimental studies (2) on women with a history of stage I-III breast cancer diagnosis, (3) focusing on the effect of dietary interventions (alone or in combination with physical activity recommendations) on QoL. Unpublished studies were searched and the reference lists from selected articles were examined to identify additional studies. The Preferred Reporting Items for Systematic Reviews and Meta-Analyses (PRISMA) guidelines were followed [44].

#### 2.2.2. Data Extraction

From all the selected articles, two of the authors (AM and RMSL) independently extracted the following information using a standard format: first author’s last name, year of publication, study design, country where the study was performed, ethnicity, number of participants and method for QoL assessment. The authors also summarized the main findings on the effects of dietary intervention, alone or in combination with other recommendations, on QoL and secondary outcomes (changes in weight status, dietary habits and physical activity). 

#### 2.2.3. Risk of bias assessment

For randomized controlled trials included in the systematic review, two of the authors (AM and RMSL) also evaluated the risk of bias using the Cochrane’s Collaboration tool [45]. For each study, a score (‘low risk of bias’, ‘unclear risk of bias’ or ‘high risk of bias’) was assigned to the following items: random sequence generation; concealment of the allocation sequence; blinding of outcome assessment; incomplete outcome data; selective outcome reporting and other biases. Disagreements were resolved by discussion with a third author (A.A.). 

## 3. Results

### 3.1. Cross-Sectional Study

#### 3.1.1. Study Population and Quality of Life

In the current cross-sectional analysis, we used data from 68 stage I-III breast cancer women, aged 36-68 years. We first compared QoL of our study population with reference values from the EORTC Quality of Life Group’s Cross-Cultural Analysis Project [46]. With respect to the QLQ-C30 module, study participants reported worst scores for emotional (*p* = 0.015) and cognitive functioning (*p* = 0.023), insomnia (*p* < 0.001) and financial impact (*p* = 0.002) scales compared with the EORTC reference values. By contrast, they exhibited better scores for the loss of appetite (*p* = 0.003). However, only insomnia, financial impact and loss appetite remained significantly different from reference values after Bonferroni correction (*p*-values ≤ 0.003). Interestingly, with respect to the QLQ-BR23 module, study participants showed the worst scores for all scales than those reported by the EORTC project also after adjusting for multiple comparisons (*p*-values ≤ 0.006).

#### 3.1.2. Mediterranean Diet and Quality of Life

**Figure 1:**
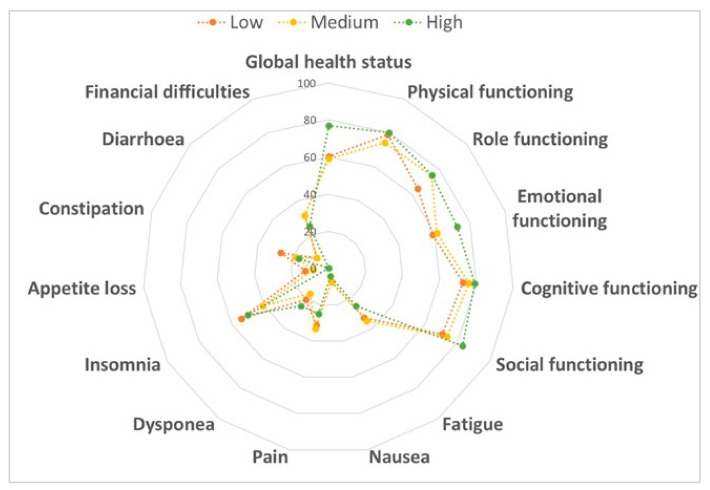
Comparison of the quality of life according to adherence to the Mediterranean diet. Data were compared using the one-way ANOVA.

In our population, we first assessed the association of adherence to MD and its typical food groups with QoL. The comparison between women who met MD criteria with who did not demonstrated the beneficial effects of several food groups: women who consumed less than one serving of red meat per day reported better scores for dyspnea (*p* = 0.035) and financial difficulties (*p* = 0.008); women who drank less than two servings of carbonated beverages per day showed better scores for dyspnea (*p* = 0.004) and insomnia (*p* = 0.016); women who drank seven or more glasses of wine per week reported a better score for sexual enjoyment (*p* = 0.025) and women who consumed two or more dishes seasoned with sofrito per week showed a better score for sexual functioning (*p* = 0.035). By contrast, we also observed the negative effects of other food groups on QoL: women who used olive oil as the main culinary fat reported worst scores for sexual functioning (*p* = 0.044) and enjoyment (*p* = 0.008); women who consumed three or more fish servings per week showed the worst scores for emotional (*p* = 0.049) and cognitive functioning (*p* = 0.034), financial difficulties (*p* = 0.034), side effects (*p* = 0.019) and breast symptoms (*p* = 0.008); women who consumed less than three servings of commercial sweets or pastries per week reported worst scores for the loss of appetite (*p* = 0.043), body image (*p* = 0.027) and arm symptoms (*p* = 0.045) and women who consumed three or more servings of nuts per week showed the worst score for role functioning (*p* = 0.004). However, no differences were evident after the correction for multiple comparisons. Similarly, the comparison across categories of adherence to MD showed that it did not affect overall QoL and its subscales (Figure 1). 

#### 3.1.3. Physical Activity and Quality of Life

**Figure 2:**
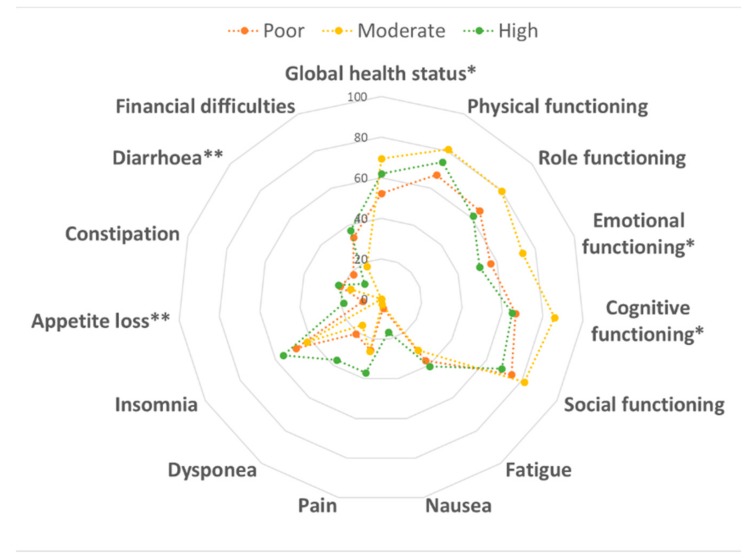
Comparison of the quality of life according to physical activity level. Data were compared using the one-way ANOVA. * *p* < 0.05 and ** *p* < 0.01.

With respect to physical activity, we first observed that 24.7% of women reported low physical activity, while 50.5% and 24.7% showed moderate or high physical activity, respectively. Figure 2 displays QLQ-C30 scores by physical activity levels. Notably, we observed better scores for emotional (*p* = 0.028) and cognitive (*p* = 0.016) functioning, loss of appetite (*p* = 0.008) and diarrhea (*p* = 0.001) among moderately physically active women than those who performed less or more physical activity. These findings resulted in better global health status in women who performed moderate physical activity (*p* = 0.035). However, only diarrhea subscale remained significantly better among moderately physically active women after Bonferroni correction (*p* ≤ 0.003). With respect to the QLQ-BR23 module, no differences were evident.

#### 3.1.4. Weight Status and Quality of Life

**Figure 3:**
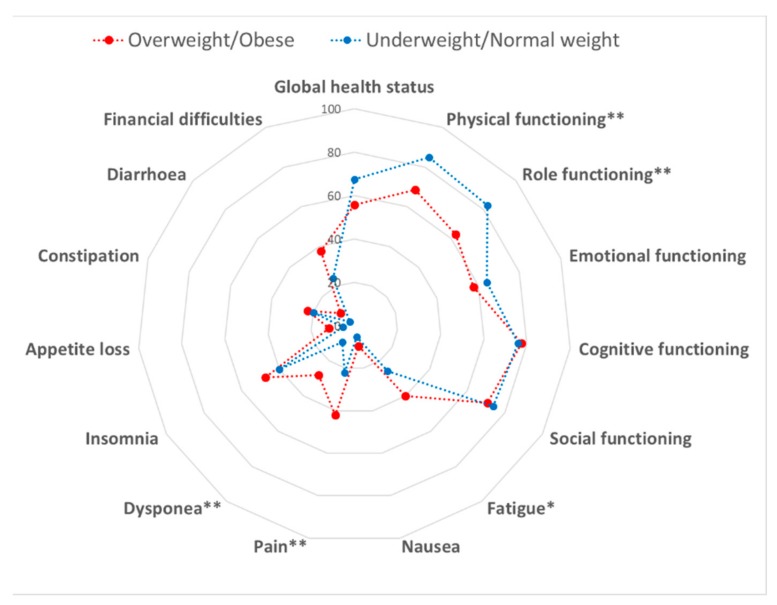
Comparison of the quality of life between overweight/obese and underweight/normal weight women. Data were compared using the Student’s *t*-test. * *p* < 0.05 and ** *p* < 0.01.

We finally evaluated the association between the weight status and QoL. According to BMI (mean = 26.4 kg/m^2^; SD = 4.8 kg/m^2^), we identified 0.7% underweight, 44.9% normal weight, 33.1% overweight and 21.3% obese women. We first observed that several QoL sub-scores (i.e., physical and role functioning, fatigue, nausea, pain, dyspnea, insomnia, appetite loss, constipation, diarrhea and financial difficulties) decreased from underweight to obese women (*p*-values < 0.05). Particularly, the comparison between underweight/normal weight and overweight/obese women demonstrated worst scores for physical (*p* = 0.001) and role functioning (*p* = 0.004), fatigue (*p* = 0.025), pain (*p* = 0.009) and dyspnea (*p* = 0.003) in the latter group (Figure 3). However, only physical functioning and dyspnea remained significantly better among underweight/normal weight women after Bonferroni correction (*p*-values ≤ 0.003).

Similar results were obtained with respect to the QLQ-BR23 module, no differences by weight status were evident.

### 3.2. Findings from the Systematic Review

**Figure 4:**
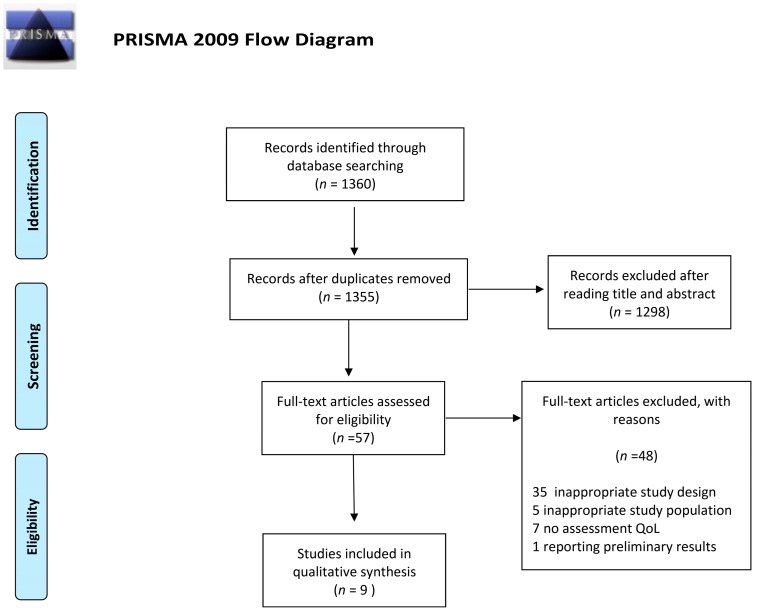
Preferred reporting items for literature search (PRISMA) flow diagram of the study selection.

The detailed steps of the study selection are given as a PRISMA flow diagram in Figure 4. The current systematic review finally included nine experimental studies (Table 1). In particular, we included studies from Europe (*n* = 4), America (*n* = 4) and Asia (*n* = 1), and different tools for assessing QoL in breast cancer survivors were reported. Particularly, one study analyzed data from the QLQ-C30 and the SF-36 modules at the same time, while five used only the QLQ-C30 or the SF-36. Moreover, two studies collected data from the FACT module and one from other less frequently used scales. With respect to study design, we included six randomized controlled trials, two single-arm trials and one randomized cross-over pilot study. Duration of the intervention ranged from 2 weeks to 12 months. In general, all the studies demonstrated significant improvements in overall QoL and/or its subscales after the interventions. However, differences in the study design, interventions and tools used for QoL assessment did not allow us to provide an overall estimate. Moreover, only the single-arm trial by Bauersfeld and colleagues evaluated the effect of an intervention based exclusively on dietary recommendations. They demonstrated that short-term fasting followed by normo-caloric diet counteracted the reduction of QoL in the first half of chemotherapy [47]. Specifically, the reduction in QoL after chemotherapy was less than the minimally important difference with short-term fasting but greater than the minimally important difference for non-fasted periods.

The remaining studies, instead, proposed several combined interventions to promote a healthy diet and regular physical activity among breast cancer survivors, including stage-matched telephone counseling [48], automated prompts [49], physical and nutritional interventions in hydrothermal centers [50] and weight loss programs [51,52]. Befort and colleagues conducted a single-arm trial on 34 obese stage I-III breast cancer survivors that were instructed to follow dietary (i.e., consuming ≥5 fruit and vegetable servings per day, prepackaged frozen entrees and shakes) and physical activity (i.e., 225 minutes per week of moderate intensity activity) recommendations [53]. This intervention significantly improved several QoL domains, such as mood, body image and sexuality. The authors also observed significant improvements in weight, waist circumference, daily energy intake, fruit and vegetables consumption and physical activity level [53]. The randomized controlled trial by Demark-Wahnefried and colleagues confirmed the efficacy of recommendations on diet and physical activity for improving QoL among overweight or obese breast cancer survivors (*n* = 692) [54]. In particular, the intervention consisted of written instructions to follow the ACS guidelines on weight control, diet and physical activity. From baseline to 6 months, physical function decreased and symptoms increased in controls but not in the intervention arm. Improvements in vitality were evident in both groups, but greater in the intervention arm. By contrast, depressive symptoms increased in the intervention arm and became significant after 24 months [54]. The randomized controlled trial by Kim and colleagues evaluated the effects of a telephone counseling with personal prescription for regular exercise and balanced diet based on ACS guidelines. They reported that the intervention group showed significantly improvement in emotional functioning, fatigue and depression than the control arm [48]. Interestingly, the authors also reported that the intervention increased motivational readiness for exercise and diet. Similarly, Kwiatkowski and colleagues conducted a randomized controlled trial evaluating the effect of a two-week intervention in hydrothermal centers [50]. The intervention, consisting of a daily group supervised physical training, dietary education, physiotherapy and psychological support, significantly improved QoL after 6 and 12 months, with greater improvements in mental and physical sub-scores [50]. In line with these findings, the randomized controlled trial by Morey and colleagues delivered a 12-month diet and exercise intervention via telephone counseling and tailored mailed materials; the control group consisted of a delayed-intervention control arm [49]. The authors demonstrated a significant improvement in physical activity, dietary behaviors and overall QoL in the intervention arm. Moreover, after 12 months, the mean function scores declined less rapidly in the intervention group compared with the controls group [49]. The randomized controlled trial by Travier and colleagues evaluated the effect of a 12-week intervention based on dietary and physical activity recommendations, with the aim of promoting weight loss [52]. Participants who completed the intervention reported significant improvements in QoL and its sub-scores. In addition, they also reported beneficial effects on weight loss and cardiorespiratory fitness [52]. Similarly, two independent research groups aimed to investigate beneficial effects of interventions based on aerobic exercise and dietary counseling [55,56]. Ghavami and colleagues noted that a 24-week intervention significantly improved symptom relief, functional and global health status [56]. In line, Swisher and colleagues observed significant improvements in overall physical wellbeing, as well as in breast-cancer specific subscales and total score. The intervention also reduced body fat and sedentary time [55].

### 3.3. Risk of Bias in Randomized Controlled Trials

Risk-of-bias assessment in randomized controlled trials was shown in Tables S1–S3. In general, we identified low risk of selection bias due to random sequence generation and allocation concealment. In four studies, we noted an unclear risk of detection bias due to insufficient information on the blinding of outcome assessment. For the other domains (i.e., attrition, reporting and other bias), low risk was evident.

## 4. Discussion

The first aim of our work was to investigate the potential effect of MD on the QoL of stage I-III breast cancer patients who completed radiotherapy or chemotherapy treatment at least 6 months prior to the recruitment. For doing that, we conducted a cross-sectional analysis showing that low consumption of red meat and carbonated beverages, daily consumption of wine and high consumption of dishes seasoned with sofrito were associated with better scores for several QoL subscales. This was partially in line with previous studies reporting better scores in some QoL subscales—specifically global health, cognitive functioning and dyspnea—among women who adhered to healthy dietary patterns [20,22]. Other studies, however, demonstrated a positive effect of healthy diet on depressive symptoms [19], but no direct correlation with the overall QoL was evident [57]. Accordingly, in our study population, we failed in demonstrating an association between adherence to MD and QoL. Indeed, MD is also characterized by some typical products—for example, olive oil, fish and nuts—that seemed to have a negative effect on several QoL subscales. The low sample size should be considered when interpreting our results. In fact, we could not adjust for potential confounders (e.g., age and other social and demographic factors) nor evaluate a mediating effect. As stated by Lua and colleagues, the relationship between diet and QoL could be mediated by other behavioral and clinical factors, such as an engagement in physical activity and weight control [57]. In our study, we observed worst scores for physical and role functioning, fatigue, pain, dyspnea among overweight or obese women compared with their normal weight counterpart. With regards to exercise, we noted the most positive effect on QoL among women who performed moderate physical activity, with better scores for global health status, emotional and cognitive functioning, loss of appetite and diarrhea. Several observational studies already demonstrated benefits from more engagement in physical activity on the QoL of breast cancer survivors [25,26,27]. More recently, a lot of research groups aimed to demonstrate the beneficial effects of promoting exercise and avoiding sedentary lifestyles among breast cancer survivors. Interestingly, a meta-analysis of thirty-three randomized controlled trials concluded that QoL was significantly improved in the exercise intervention group. Besides, exercise was also associated with positive outcomes in BMI, lean mass and muscle strength, as well as in the serum concentration of insulin, insulin-like growth factor-II and insulin-like growth factor binding protein-1 [58].

While benefits of physical activity are already well known, findings about a potential effect of dietary intervention are still inconclusive. Thus, we also conducted a systematic review of experimental studies investigating the effect of dietary interventions, alone or in combination with other recommendations, on the QoL of breast cancer survivors. Although all the studies underlined significant improvements in QoL after the interventions, only a trial evaluated the sole and exclusive effect of dietary intervention [47]. The rest of studies, instead, combined the promotion of dietary recommendations with programs aiming to increase exercise and physical activity among breast cancer survivors. Beyond that, differences in study design and in tools used for QoL assessment did not allow us to provide an overall estimate. What was certain was that QoL increased with increasing number of lifestyle recommendations, especially with those proposed by the ACS for cancer survivors.

## 5. Conclusions

Our study confirmed that more efforts are needed to understand the exclusive effect of diet and dietary interventions on the QoL of breast cancer survivors. Instead, evidence of benefits from physical activity and weight management is already well established. However, our results raise the need for understanding whether tackling sedentary habits represents the best strategy to improve QoL instead of promoting intensive exercise. To fill these gaps, further research on the effects of physical activity and healthy diet on QoL among breast cancer survivors should be based on more homogenous methods, larger population-based studies and further randomized controlled trials, which might allow us to evaluate the interactions of healthy behaviors and to improve the robustness of current evidence.

## Figures and Tables

**Table 1:** Characteristics of experimental studies included in the systematic review.

Author, Year	Country	Study Design	Population Characteristics	Intervention	QoL Assessment	Main Findings
Bauersfeld, 2018 [47]	Germany	Randomized cross-over pilot study	30 breast cancer patients with scheduled chemotherapy	Short-term fasting followed by normo-caloric diet or vice versa in the first half of chemotherapy	FACT-G and FACT-F	The reduction in QoL after chemotherapy was less than the minimally important difference with short-term fasting but greater than the minimally important difference for non-fasted periods (*p* < 0.05). There were no serious adverse effects of intervention
Befort, 2011 [53]	USA	Single-arm trial	34 obese stage I–III breast cancer survivors	Participants were instructed to follow a diet with ≥5 fruit and vegetable servings per day, prepackaged frozen entrees, and shakes. They were also instructed to gradually increase their physical activity to 225 minutes per week of moderate intensity activity	Breast Cancer Prevention Trial Symptom Checklist, PHQ-9, and the 32-item Body Image and Relationships scale	Significant improvements were evident for several QoL domains, including joint pain (*p* < 0.001), depression (*p* = 0.001), depression (*p* = 0.001), strength and health (*p* < 0.001), social barriers (*p* = 0.02) and sexuality (*p* < 0.001). The intervention also significantly reduced weight, waist circumference, daily energy intake and increased fruit and vegetables consumption and physical activity level
Demark-Wahnefried, 2015 [54]	USA	Randomized controlled trial	692 obese or overweight women with a history of stage I–III breast cancer diagnosis who completed treatment	Intensive program consisting of six months of group sessions, personalized guidance via telephone and/or email to reduce weight and adhere to dietary and physical activity guidelines of the American Cancer Society. Controls received two contacts, one at baseline and another at six months	SF-36	From baseline to 6 months, significant decreases in physical function (*p* = 0.01) and increases in symptoms (*p* = 0.002) were observed among controls, but not in the intervention arm. Improvements in vitality were evident in both arms, with greater improvement in the intervention arm (*p* = 0.05). By contrast, depressive symptoms increased in the intervention arm and became significant after 24 months (*p* = 0.031)
Kim, 2011 [48]	Korea	Randomized controlled trial	45 women with breast cancer who completed treatment	Telephone counseling with a personal prescription for regular exercise and balanced diet programs based on the guidelines for cancer survivors	QLQ-C30	Compared with controls, the intervention group reported greater improvement in emotional functioning (*p* = 0.004), fatigue (*p* = 0.001) and depression (*p* = 0.035), and also in motivational readiness for exercise (*p* = 0.006) and diet (*p* < 0.001)
Kwiatkowski, 2017 [50]	France	Randomized controlled trial	251 breast cancer patients post chemotherapy	Two-week intervention in hydrothermal center, consisting of daily group supervised physical training, dietary education, physiotherapy and psychological support	SF-36	The intervention improved QoL at 6 and 12 months (*p* < 0.001; *p* = 0.032), with greater improvements in mental and physical sub-scores (*p*-values < 0.001)
Morey, 2009 [49]	USA	Randomized controlled trial	143 breast cancer patients	12-month diet and exercise intervention delivered via telephone counseling and tailored mailed materials versus a delayed-intervention control arm	SF-36	The intervention significantly improved physical activity, dietary behaviors, and overall QoL (*p*-values < 0.05). At 12 months, the mean function scores declined less rapidly in the intervention group compared with the control group (*p* = 0.03).
Travier, 2015 [52]	Spain	Phase II single-arm trial	37 breast cancer survivors who had completed chemotherapy and/or radiotherapy	12-week intervention including dietary and physical activity recommendations, with the aim of promoting weight loss	QLQ-C30 and SF-36	Participants who completed the intervention reported significant improvements in QoL (*p* < 0.001) and its sub-scores (*p*-values < 0.05), and also in weight, BMI and cardiorespiratory fitness
Ghavami, 2017 [56]	Turkey	Randomized controlled trial	40 breast cancer survivors with stage I–III breast cancer and 40 control	Practice supervised aerobic exercises with dietary energy restriction training for 24 weeks	QLQ-C30	Compared with controls, the intervention significantly improved symptom relief (*p* < 0.001), functional (*p* < 0.001) and global health status (*p* < 0.001)
Swisher, 2015 [55]	USA	Randomized controlled trial	Survivors of triple-negative breast cancer:13 intervention, 10 control	Supervised, moderate-intensity aerobic exercise (150 min per week, for 12 weeks) and diet counseling (two individual sections with a dietitian)	FACT-B	Compared with control, the intervention significantly improved overall physical wellbeing (*p* < 0.05), and breast-cancer specific subscales (*p* < 0.01) and total score (*p* < 0.05). The intervention also reduced body fat and sedentary time

Abbreviations: QoL, Quality of life; QLQ-C30, European Organization for the Research and Treatment of Cancer Quality-of-Life Questionnaire–Core 30; QLQ-BR23, Quality of Life Questionnaire Breast Cancer Module 23; SF-36, Short form-36; FACT, Functional Assessment of Cancer Therapy; PHQ-9, Patient Health Questionnaire.

## Supplementary Materials

The following are available online at https://www.mdpi.com/2072-6694/12/2/322/s1, Table S1: Criteria for positive items of the Mediterranean Diet Assessment Tool (Martinez-Gonzalez et al., 2002), Table S2: PRISMA checklist of the systematic review, Table S3: Risk of bias assessment of randomized controlled trials included in the systematic review.
