# Supplementary material for: The Effects of Diet and Dietary Interventions on the Quality of Life among Breast Cancer Survivors: A Cross-Sectional Analysis and a Systematic Review of Experimental Studies
Source: Cancers (Basel). 2020 Jan 30;12(2):322. doi: 10.3390/cancers12020322 (PMC7072135; doi:10.3390/cancers12020322)
Supplement: Supplementary file 1 [file cancers-12-00322-s001.pdf]

Supplementary Materials: The Effects of Diet and Dietary Interventions on Quality of Life among Breast Cancer Survivors: A Cross-sectional Analysis and a Systematic Review of Experimental Studies

Martina Barchitta, Andrea Maugeri, Roberta Magnano San Lio, Annalisa Quattrocchi, Flori Degrassi, Francesca Catalano, Guido Basile and Antonella Agodi

Table S1. Criteria for positive items of the Mediterranean Diet Assessment Tool (Martinez-Gonzalez et al., 2002) [1].

Items	Criteria
1	Using olive oil as main culinary fat
2	≥ 4 tablespoon of olive oil in a given day (including oil used for frying, salads, out-of-house meals)
3	≥ 2 (≥ 1 portions raw or as a salad) vegetable servings per day (1 serving: 200 g)
4	≥ 3 fruit units (including natural fruit juices) per day
5	< 1 serving of red meat, hamburger, or meat products (ham, sausage) per day (1 serving: 100-150 g)
6	< 1 serving of butter, margarine, or cream per day (1 serving: 12 g)
7	< 1 sweet of carbonated beverages per day
8	≥ 7 glasses of wine per week
9	≥ 3 servings of legumes per week (1 serving: 150 g)
10	≥ 3 servings of fish or shellfish per week (1 serving: 100–150 of fish or 200 g of shellfish)
11	< 3 times per week consuming commercial sweets or pastries (not homemade)
12	≥ 3 servings of nuts (including peanuts) per week (1 serving: 30 g)
13	Preferable consumption of chicken, turkey or rabbit meat - instead of pork, hamburger of sausage
14	≥ 2 times per week consuming vegetables, pasta, rice or other dishes seasoned with sofrito (sauce made with tomato and onion, leek, or garlic and simmered with olive oil)

Table S2. PRISMA checklist of the systematic review.

Section/topic	Number of item	Checklist item	Reporting page
		**TITLE**	
Title	1	Identify the report as a systematic review, meta-analysis, or both.	1
		**ABSTRACT**	
Structured summary	2	Provide a structured summary including, as applicable: background; objectives; data sources; study eligibility criteria, participants, and interventions; study appraisal and synthesis methods; results; limitations; conclusions and implications of key findings; systematic review registration number.	1
		**INTRODUCTION**	
Rationale	3	Describe the rationale for the review in the context of what is already known.	2
Objectives	4	Provide an explicit statement of questions being addressed with reference to participants, interventions, comparisons, outcomes, and study design (PICOS).	2
		**METHODS**	
Protocol and registration	5	Indicate if a review protocol exists, if and where it can be accessed (e.g., Web address), and, if available, provide registration information including registration number.	NA
Eligibility criteria	6	Specify study characteristics (e.g., PICOS, length of follow-up) and report characteristics (e.g., years considered, language, publication status) used as criteria for eligibility, giving rationale.	4

Information sources	7	Describe all information sources (e.g., databases with dates of coverage, contact with study authors to identify additional studies) in the search and date last searched.	4
Search	8	Present full electronic search strategy for at least one database, including any limits used, such that it could be repeated.	4
Study selection	9	State the process for selecting studies (i.e., screening, eligibility, included in systematic review, and, if applicable, included in the meta-analysis).	4
Data collection process	10	Describe method of data extraction from reports (e.g., piloted forms, independently, in duplicate) and any processes for obtaining and confirming data from investigators.	4
Data items	11	List and define all variables for which data were sought (e.g., PICOS, funding sources) and any assumptions and simplifications made.	4
Risk of bias in individual studies	12	Describe methods used for assessing risk of bias of individual studies (including specification of whether this was done at the study or outcome level), and how this information is to be used in any data synthesis.	4
Summary measures	13	State the principal summary measures (e.g., risk ratio, difference in means).	NA
Synthesis of results	14	Describe the methods of handling data and combining results of studies, if done, including measures of consistency (e.g., I^2) for each meta-analysis.	NA
Risk of bias across studies	15	Specify any assessment of risk of bias that may affect the cumulative evidence (e.g., publication bias, selective reporting within studies).	4
Additional analyses	16	Describe methods of additional analyses (e.g., sensitivity or subgroup analyses, meta-regression), if done, indicating which were pre-specified.	NA
		**RESULTS**	
Study selection	17	Give numbers of studies screened, assessed for eligibility, and included in the review, with reasons for exclusions at each stage, ideally with a flow diagram.	6, Figure 3
Study characteristics	18	For each study, present characteristics for which data were extracted (e.g., study size, PICOS, follow-up period) and provide the citations.	Table 1
Risk of bias within studies	19	Present data on risk of bias of each study and, if available, any outcome level assessment (see item 12).	9, Supplementary file
Results of individual studies	20	For all outcomes considered (benefits or harms), present, for each study: (a) simple summary data for each intervention group (b) effect estimates and confidence intervals, ideally with a forest plot.	6, Table 1
Synthesis of results	21	Present results of each meta-analysis done, including confidence intervals and measures of consistency.	NA
Risk of bias across studies	22	Present results of any assessment of risk of bias across studies (see Item 15).	9
Additional analysis	23	Give results of additional analyses, if done (e.g., sensitivity or subgroup analyses, meta-regression [see Item 16]).	NA
		**DISCUSSION**	
Summary of evidence	24	Summarize the main findings including the strength of evidence for each main outcome; consider their relevance to key groups (e.g., healthcare providers, users, and policy makers).	11
Limitations	25	Discuss limitations at study and outcome level (e.g., risk of bias), and at review-level (e.g., incomplete retrieval of identified research, reporting bias).	11
Conclusions	26	Provide a general interpretation of the results in the context of other evidence, and implications for future research.	11
		**FUNDING**	
Funding	27	Describe sources of funding for the systematic review and other support (e.g., supply of data); role of funders for the systematic review.	NA

From: Moher D, Liberati A, Tetzlaff J, Altman DG, The PRISMA Group (2009). Preferred Reporting Items for Systematic Reviews and Meta-Analyses: The PRISMA Statement. PLoS Med 6(7): e1000097. doi:10.1371/journal.pmed1000097 [2]

Table S3. Risk of bias assessment of randomized controlled trials included in the systematic review.

First Author, Year	Random Sequence Generation (Selection Bias)	Allocation Concealment (Selection Bias)	Blinding of Outcome Assessment (Detection Bias)	Incomplete Outcome Data (Attrition Bias)	Selective Reporting (Reporting Bias)	Other Bias
Demark-Wahnefried 2015 [3]	Low risk (computer random number generator)	Low risk (central allocation)	Low risk (Outcomes assessed by Investigators blind to original treatment)	Low risk (Reasons for missing outcome data unlikely to be related to true outcome)	Low risk (All outcomes reported)	Low risk (The study appears to be free of other sources of bias)
Kim 2011 [4]	Low risk (random numbers Table)	Low risk (central allocation)	Unclear risk (Insufficient information)	Low risk (Reasons for missing outcome data unlikely to be related to true outcome)	Low risk (All outcomes reported)	Low risk (The study appears to be free of other sources of bias)
Kwiatkowski 2017 [5]	Low risk (computer random number generator)	Low risk (central allocation)	Unclear risk (Insufficient information)	Low risk (Reasons for missing outcome data unlikely to be related to true outcome)	Low risk (All outcomes reported)	Low risk (The study appears to be free of other sources of bias)
Morey 2009 [6]	Low risk (computer random number generator)	Low risk (central allocation)	Low risk (Outcomes assessed by Investigators blind to original treatment)	Low risk (Reasons for missing outcome data unlikely to be related to true outcome)	Low risk (All outcomes reported)	Low risk (The study appears to be free of other sources of bias)
Ghavami 2017 [7]	Low risk (random numbers Table)	Low risk (central allocation)	Unclear risk (Insufficient information)	Low risk (Reasons for missing outcome data unlikely to be related to true outcome)	Low risk (All outcomes reported)	Low risk (The study appears to be free of other sources of bias)
Swisher 2015 [8]	Low risk (computer random number generator)	Low risk (central allocation)	Unclear risk (Insufficient information)	Low risk (Reasons for missing outcome data unlikely to be related to true outcome)	Low risk (All outcomes reported)	Low risk (The study appears to be free of other sources of bias)

Reference

1. Martínez-González, M.A.; Fernández-Jarne, E.; Serrano-Martínez, M.; Marti, A.; Martinez, J.A.; Martín-Moreno, J.M. Mediterranean diet and reduction in the risk of a first acute myocardial infarction: An operational healthy dietary score. *Eur. J. Nutr.* **2002**, *41*, 153–160, doi:10.1007/s00394-002-0370-6.
2. Moher, D.; Shamseer, L.; Clarke, M.; Ghersi, D.; Liberati, A.; Petticrew, M.; Shekelle, P.; Stewart, L.A.; Group, P.-P. Preferred reporting items for systematic review and meta-analysis protocols (PRISMA-P) 2015 statement. *Syst. Rev.* **2015**, *4*, 1, doi:10.1186/2046-4053-4-1.
3. Demark-Wahnefried, W.; Colditz, G.A.; Rock, C.L.; Sedjo, R.L.; Liu, J.; Wolin, K.Y.; Krontiras, H.; Byers, T.; Pakiz, B.; Parker, B.A.; et al. Quality of life outcomes from the Exercise and Nutrition Enhance Recovery and Good Health for You (ENERGY)-randomized weight loss trial among breast cancer survivors. *Breast Cancer Res. Treat.* **2015**, *154*, 329–337, doi:10.1007/s10549-015-3627-5.

4. Kim, S.H.; Shin, M.S.; Lee, H.S.; Lee, E.S.; Ro, J.S.; Kang, H.S.; Kim, S.W.; Lee, W.H.; Kim, H.S.; Kim, C.J.; et al. Randomized pilot test of a simultaneous stage-matched exercise and diet intervention for breast cancer survivors. *Oncol. Nurs. Forum.* **2011**, *38*, E97–E106, doi:10.1188/11.ONF.E97-E106.
5. Kwiatkowski, F.; Mouret-Reynier, M.A.; Duclos, M.; Bridon, F.; Hanh, T.; Van Praagh-Doreau, I.; Travade, A.; Vasson, M.P.; Jouvency, S.; Roques, C.; et al. Long-term improvement of breast cancer survivors' quality of life by a 2-week group physical and educational intervention: 5-year update of the 'PACThe' trial. *Br. J. Cancer* **2017**, *116*, 1389–1393, doi:10.1038/bjc.2017.112.
6. Morey, M.C.; Snyder, D.C.; Sloane, R.; Cohen, H.J.; Peterson, B.; Hartman, T.J.; Miller, P.; Mitchell, D.C.; Demark-Wahnefried, W. Effects of home-based diet and exercise on functional outcomes among older, overweight long-term cancer survivors: RENEW: A randomized controlled trial. *JAMA* **2009**, *301*, 1883–1891, doi:10.1001/jama.2009.643.
7. Haleh, G.; Neriman, A. Effects of a Lifestyle Interventions Program on Quality of Life in Breast Cancer Survivors. *Int. J. Hematol. Oncol.* **2017**. doi:10.4999/uhod.171734
8. Swisher, A.K.; Abraham, J.; Bonner, D.; Gilleland, D.; Hobbs, G.; Kurian, S.; Yanosik, M.A.; Vona-Davis, L. Exercise and dietary advice intervention for survivors of triple-negative breast cancer: Effects on body fat, physical function, quality of life, and adipokine profile. *Support Care Cancer* **2015**, *23*, 2995–3003, doi:10.1007/s00520-015-2667-z.

© 2020 by the authors. Licensee MDPI, Basel, Switzerland. This article is an open access article distributed under the terms and conditions of the Creative Commons Attribution (CC BY) license (http://creativecommons.org/licenses/by/4.0/).
